# Supplementary material for: Extracellular Citrate Treatment Induces HIF1α Degradation and Inhibits the Growth of Low-Glycolytic Hepatocellular Carcinoma under Hypoxia
Source: Cancers (Basel). 2022 Jul 10;14(14):3355. doi: 10.3390/cancers14143355 (PMC9315704; doi:10.3390/cancers14143355)
Supplement: Supplementary file 1 [file cancers-14-03355-s001.zip › cancers-1779276-supplementary.pptx]

## Slide 1
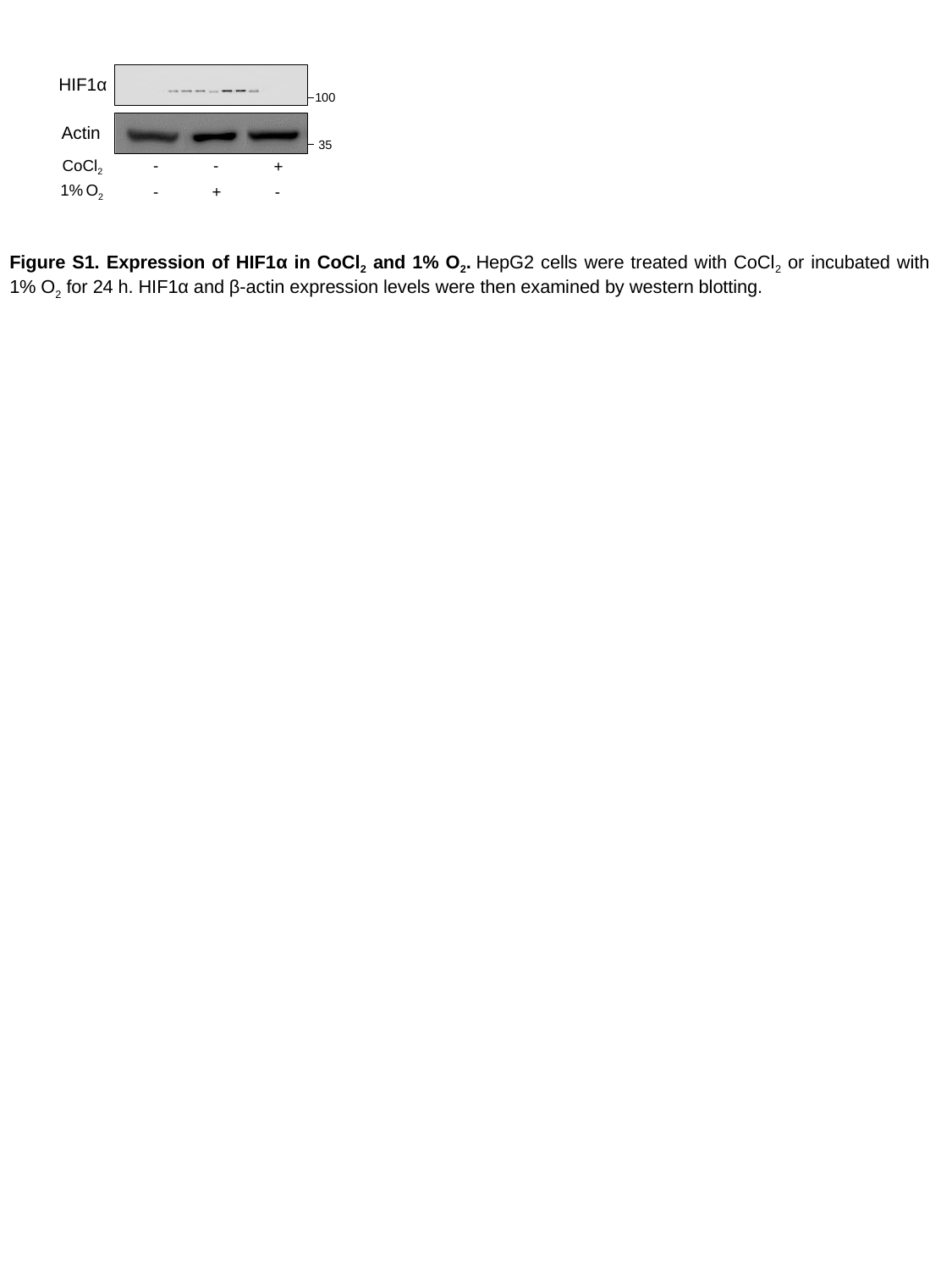

HIF1α
Actin
-
-
CoCl2
+
1% O2
-
+
-
100
35
Figure S1. Expression of HIF1α in CoCl2 and 1% O2. HepG2 cells were treated with CoCl2 or incubated with 1% O2 for 24 h. HIF1α and β-actin expression levels were then examined by western blotting.

## Slide 2
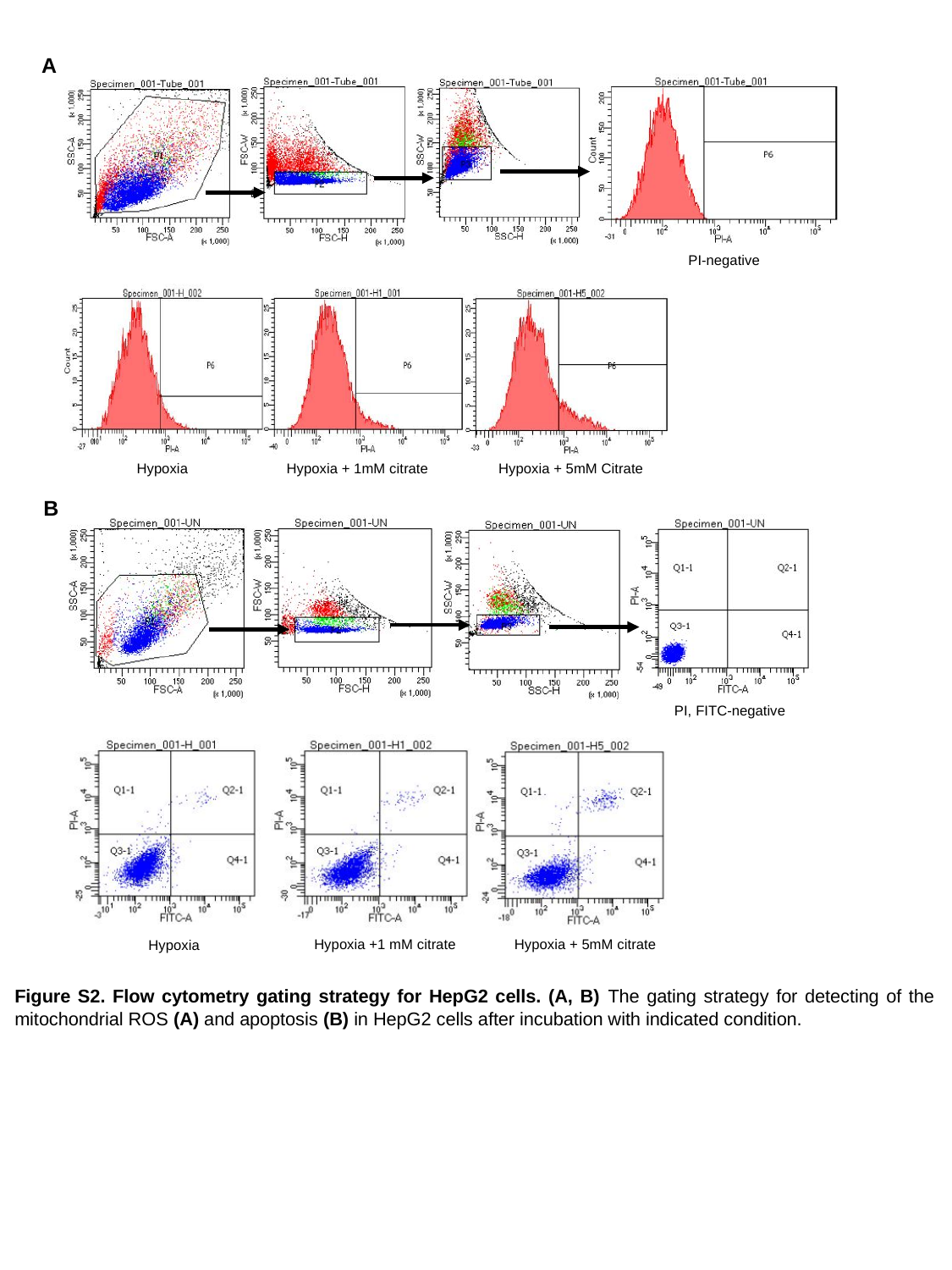

A
PI-negative
Hypoxia
Hypoxia + 1mM citrate
Hypoxia + 5mM Citrate
B
PI, FITC-negative
Hypoxia +1 mM citrate
Hypoxia + 5mM citrate
Hypoxia
Figure S2. Flow cytometry gating strategy for HepG2 cells. (A, B) The gating strategy for detecting of the mitochondrial ROS (A) and apoptosis (B) in HepG2 cells after incubation with indicated condition.

## Slide 3
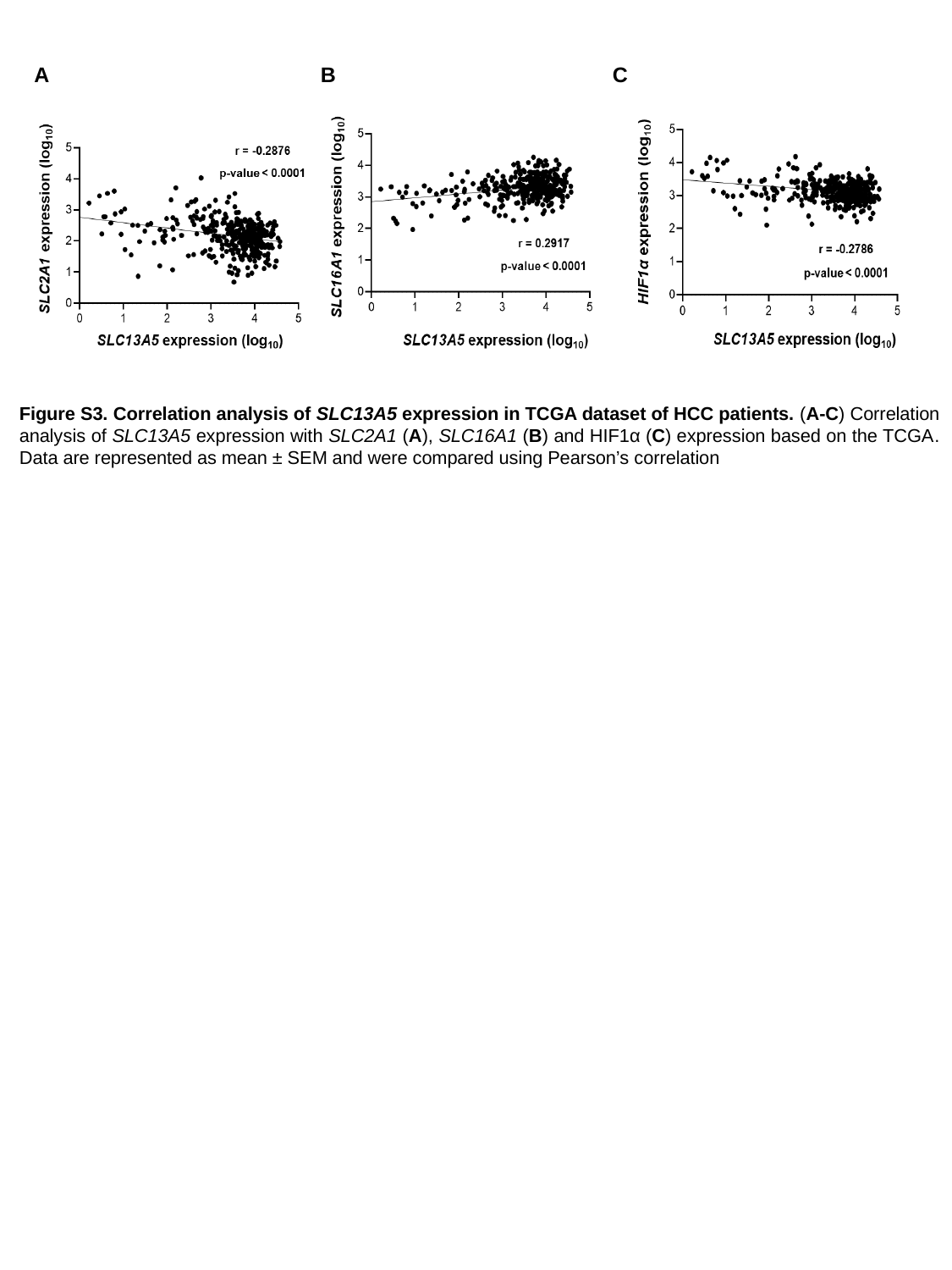

A
B
C
Figure S3. Correlation analysis of SLC13A5 expression in TCGA dataset of HCC patients. (A-C) Correlation analysis of SLC13A5 expression with SLC2A1 (A), SLC16A1 (B) and HIF1α (C) expression based on the TCGA. Data are represented as mean ± SEM and were compared using Pearson’s correlation

## Slide 4
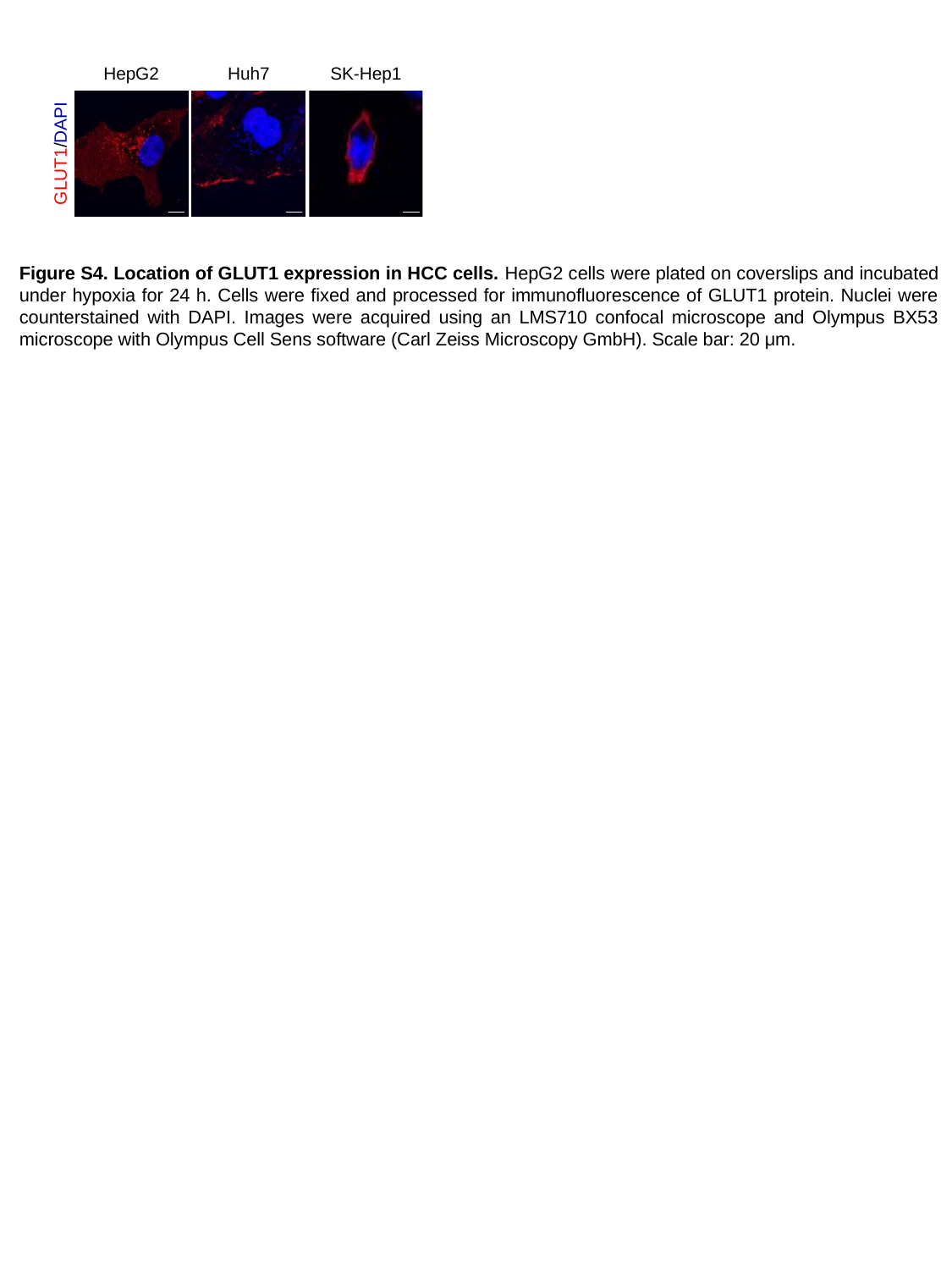

HepG2
Huh7
SK-Hep1
GLUT1/DAPI
Figure S4. Location of GLUT1 expression in HCC cells. HepG2 cells were plated on coverslips and incubated under hypoxia for 24 h. Cells were fixed and processed for immunofluorescence of GLUT1 protein. Nuclei were counterstained with DAPI. Images were acquired using an LMS710 confocal microscope and Olympus BX53 microscope with Olympus Cell Sens software (Carl Zeiss Microscopy GmbH). Scale bar: 20 μm.

## Slide 5
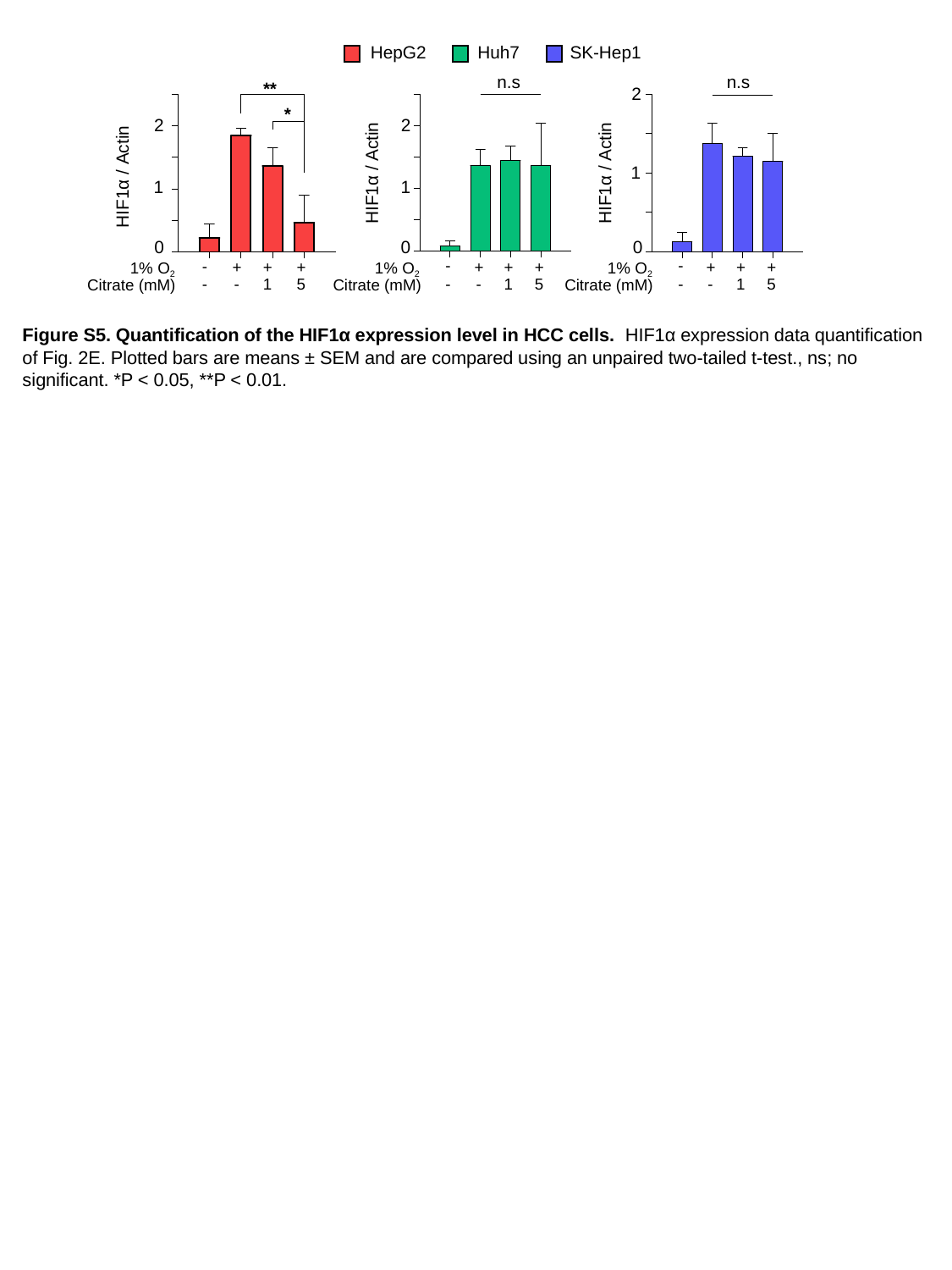

HepG2
Huh7
SK-Hep1
n.s
n.s
**
2
HIF1α / Actin
HIF1α / Actin
2
2
HIF1α / Actin
1
1
1
0
0
0
-
-
-
+
+
+
+
+
+
+
+
+
1% O2
1% O2
1% O2
-
-
1
5
-
-
1
5
-
-
1
5
Citrate (mM)
Citrate (mM)
Citrate (mM)
*
Figure S5. Quantification of the HIF1α expression level in HCC cells. HIF1α expression data quantification of Fig. 2E. Plotted bars are means ± SEM and are compared using an unpaired two-tailed t-test., ns; no significant. *P < 0.05, **P < 0.01.

## Slide 6
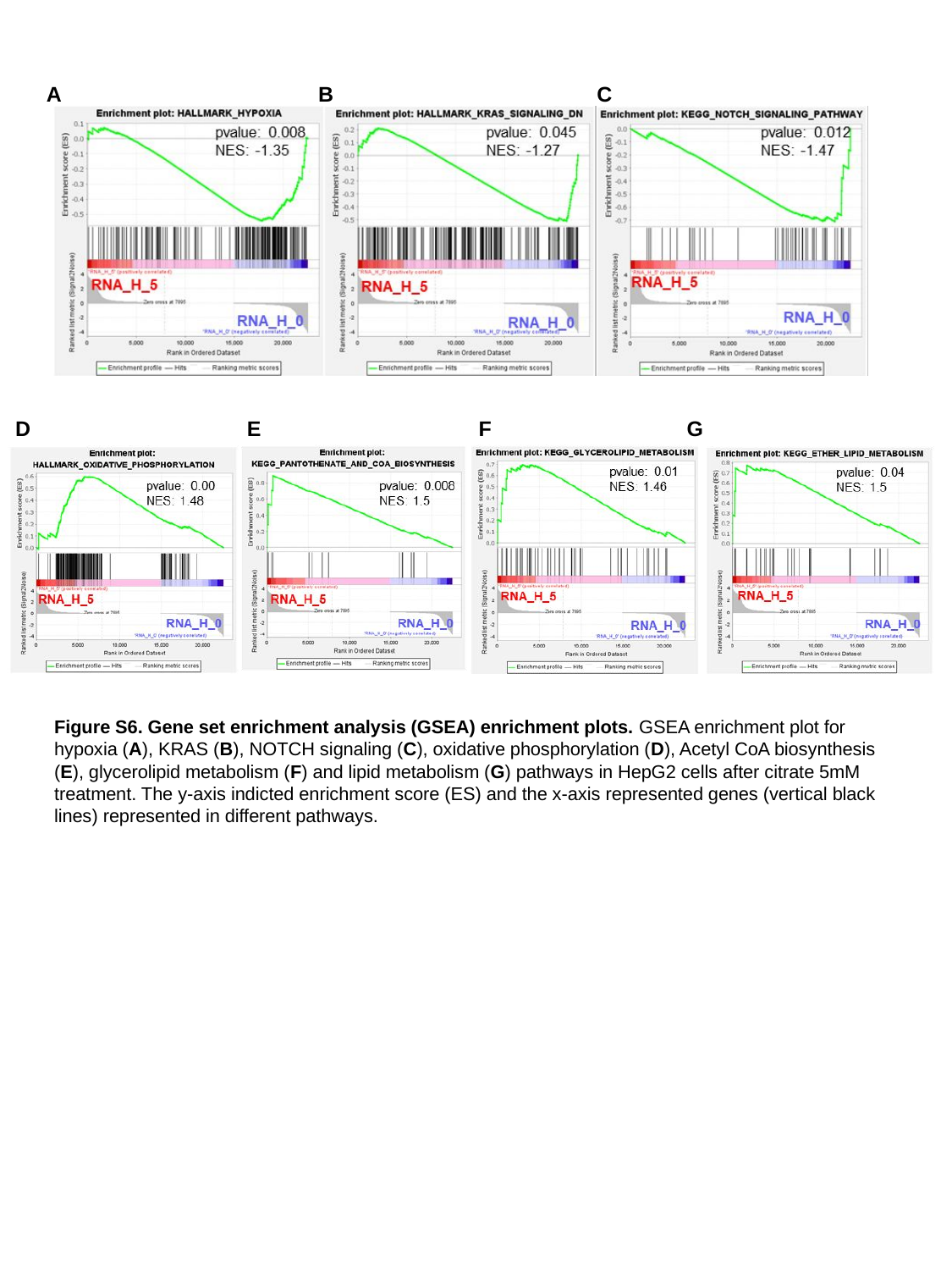

C
A
B
D
E
F
G
Figure S6. Gene set enrichment analysis (GSEA) enrichment plots. GSEA enrichment plot for hypoxia (A), KRAS (B), NOTCH signaling (C), oxidative phosphorylation (D), Acetyl CoA biosynthesis (E), glycerolipid metabolism (F) and lipid metabolism (G) pathways in HepG2 cells after citrate 5mM treatment. The y-axis indicted enrichment score (ES) and the x-axis represented genes (vertical black lines) represented in different pathways.

## Slide 7
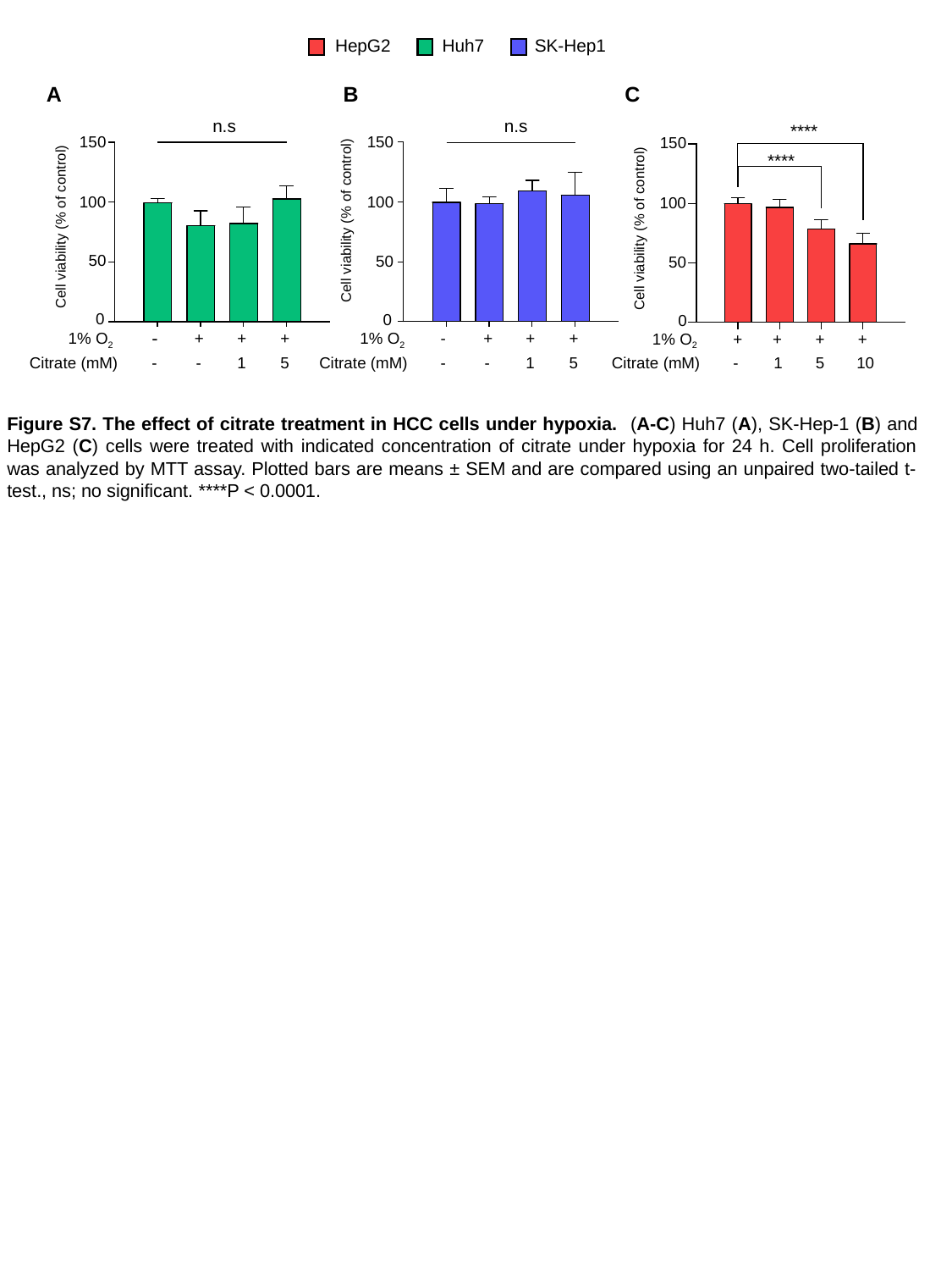

HepG2
Huh7
SK-Hep1
A
B
C
Cell viability (% of control)
n.s
n.s
Cell viability (% of control)
Cell viability (% of control)
****
150
150
150
****
100
100
100
50
50
50
0
0
0
-
1% O2
+
+
+
1% O2
-
+
+
+
1% O2
+
+
+
+
Citrate (mM)
-
-
1
5
Citrate (mM)
-
-
1
5
Citrate (mM)
-
1
5
10
Figure S7. The effect of citrate treatment in HCC cells under hypoxia. (A-C) Huh7 (A), SK-Hep-1 (B) and HepG2 (C) cells were treated with indicated concentration of citrate under hypoxia for 24 h. Cell proliferation was analyzed by MTT assay. Plotted bars are means ± SEM and are compared using an unpaired two-tailed t-test., ns; no significant. ****P < 0.0001.

## Slide 8
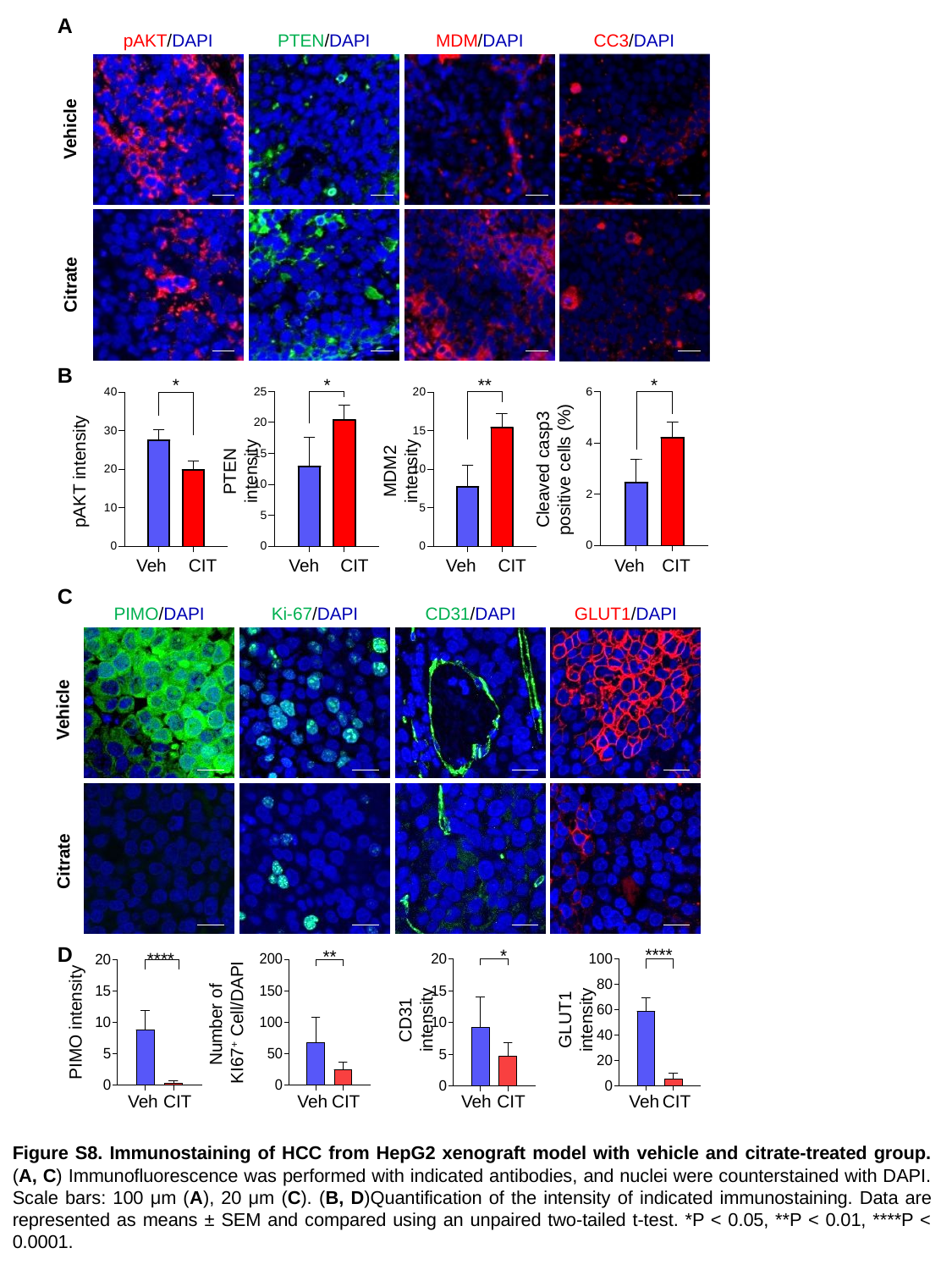

A
pAKT/DAPI
PTEN/DAPI
MDM/DAPI
CC3/DAPI
Vehicle
Citrate
B
*
*
**
*
Cleaved casp3 positive cells (%)
pAKT intensity
PTEN intensity
MDM2 intensity
Veh
CIT
Veh
CIT
Veh
CIT
Veh
CIT
C
PIMO/DAPI
Ki-67/DAPI
CD31/DAPI
GLUT1/DAPI
Vehicle
Citrate
Number of
KI67+ Cell/DAPI
****
*
**
****
GLUT1 intensity
CD31 intensity
PIMO intensity
Veh
CIT
Veh
CIT
Veh
CIT
Veh
CIT
D
Figure S8. Immunostaining of HCC from HepG2 xenograft model with vehicle and citrate-treated group. (A, C) Immunofluorescence was performed with indicated antibodies, and nuclei were counterstained with DAPI. Scale bars: 100 μm (A), 20 μm (C). (B, D)Quantification of the intensity of indicated immunostaining. Data are represented as means ± SEM and compared using an unpaired two-tailed t-test. *P < 0.05, **P < 0.01, ****P < 0.0001.

## Slide 9
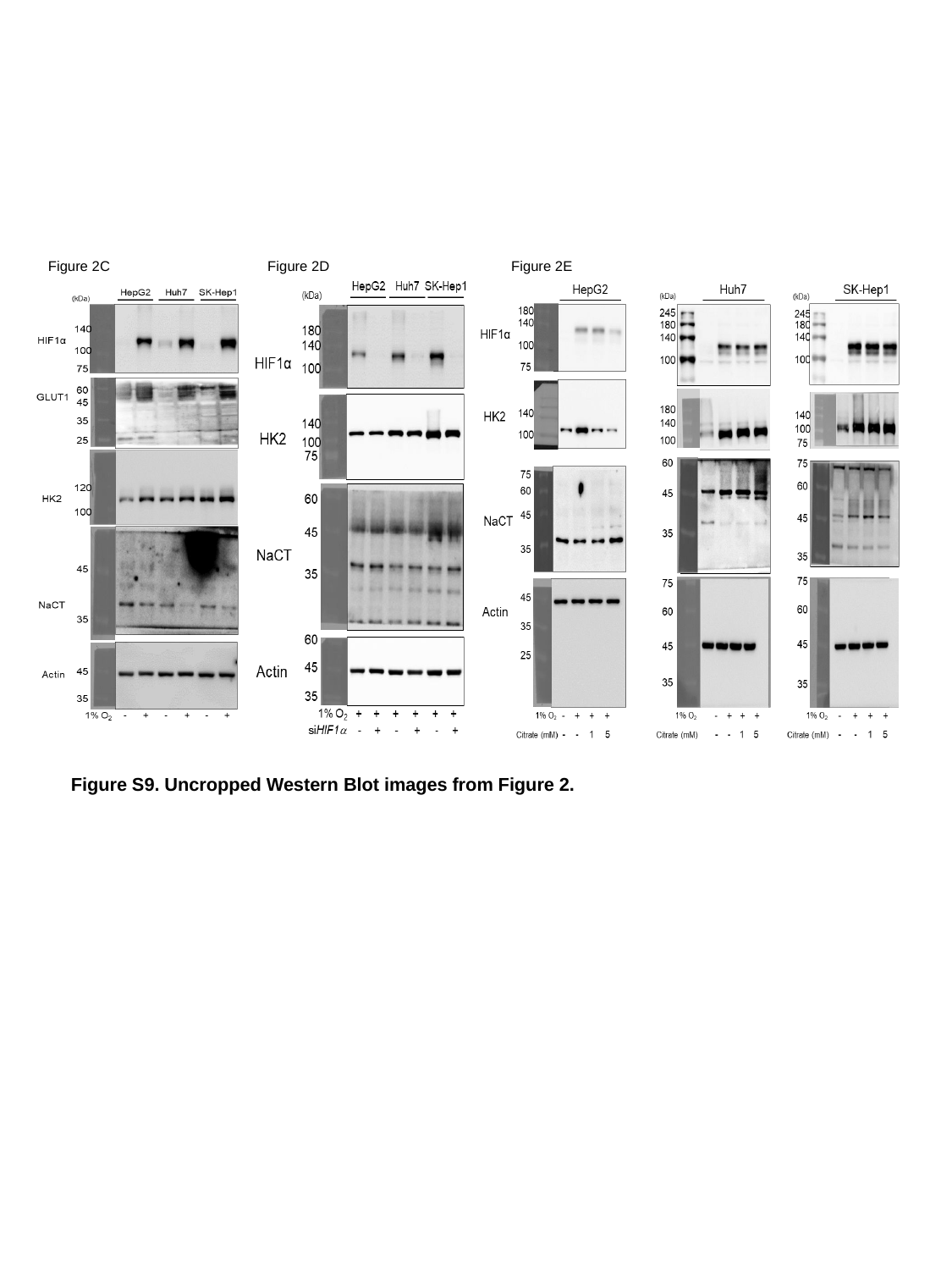

Figure 2C
Figure 2D
Figure 2E
Figure S9. Uncropped Western Blot images from Figure 2.

## Slide 10
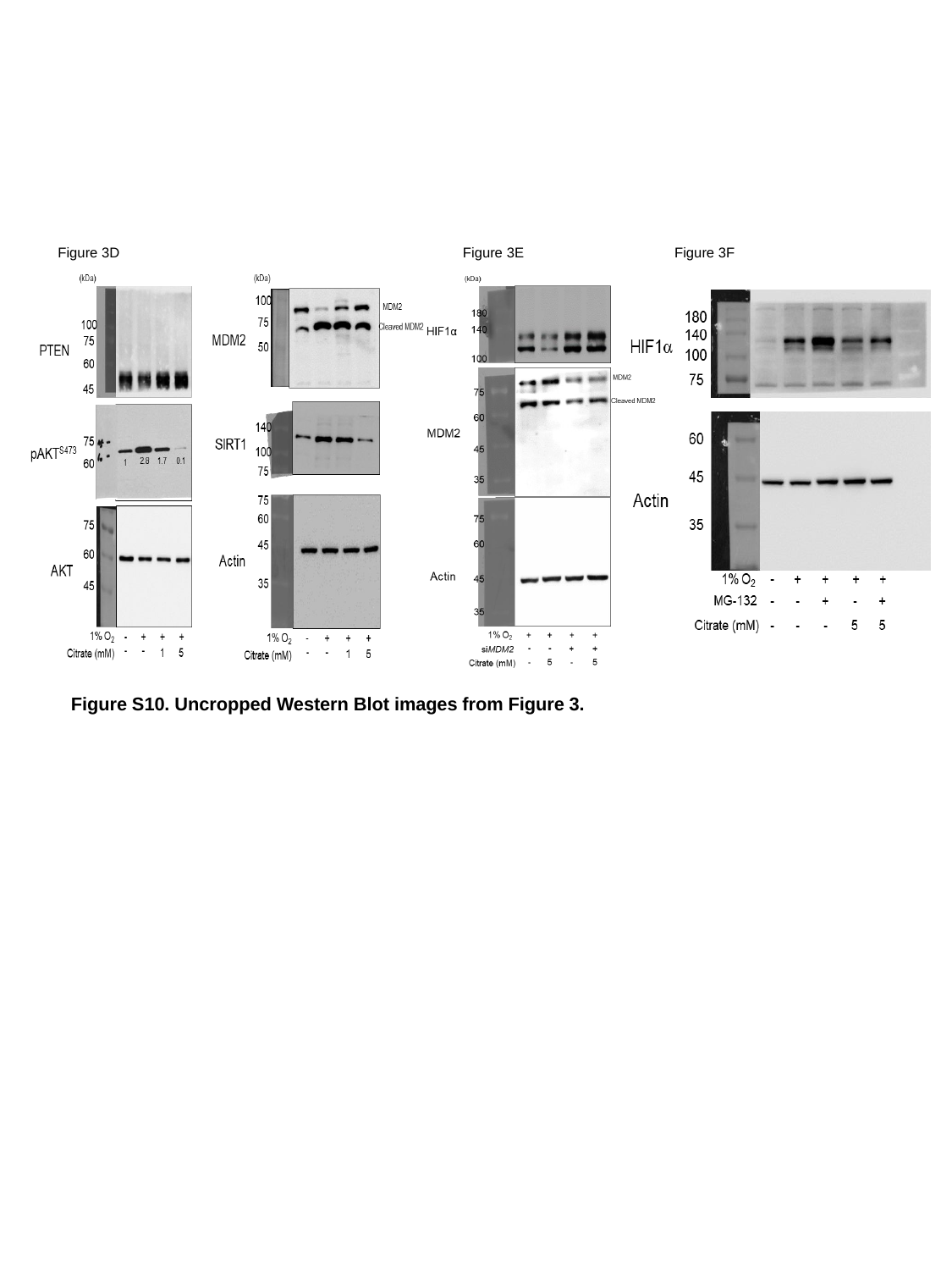

Figure 3D
Figure 3E
Figure 3F
Figure S10. Uncropped Western Blot images from Figure 3.

## Slide 11
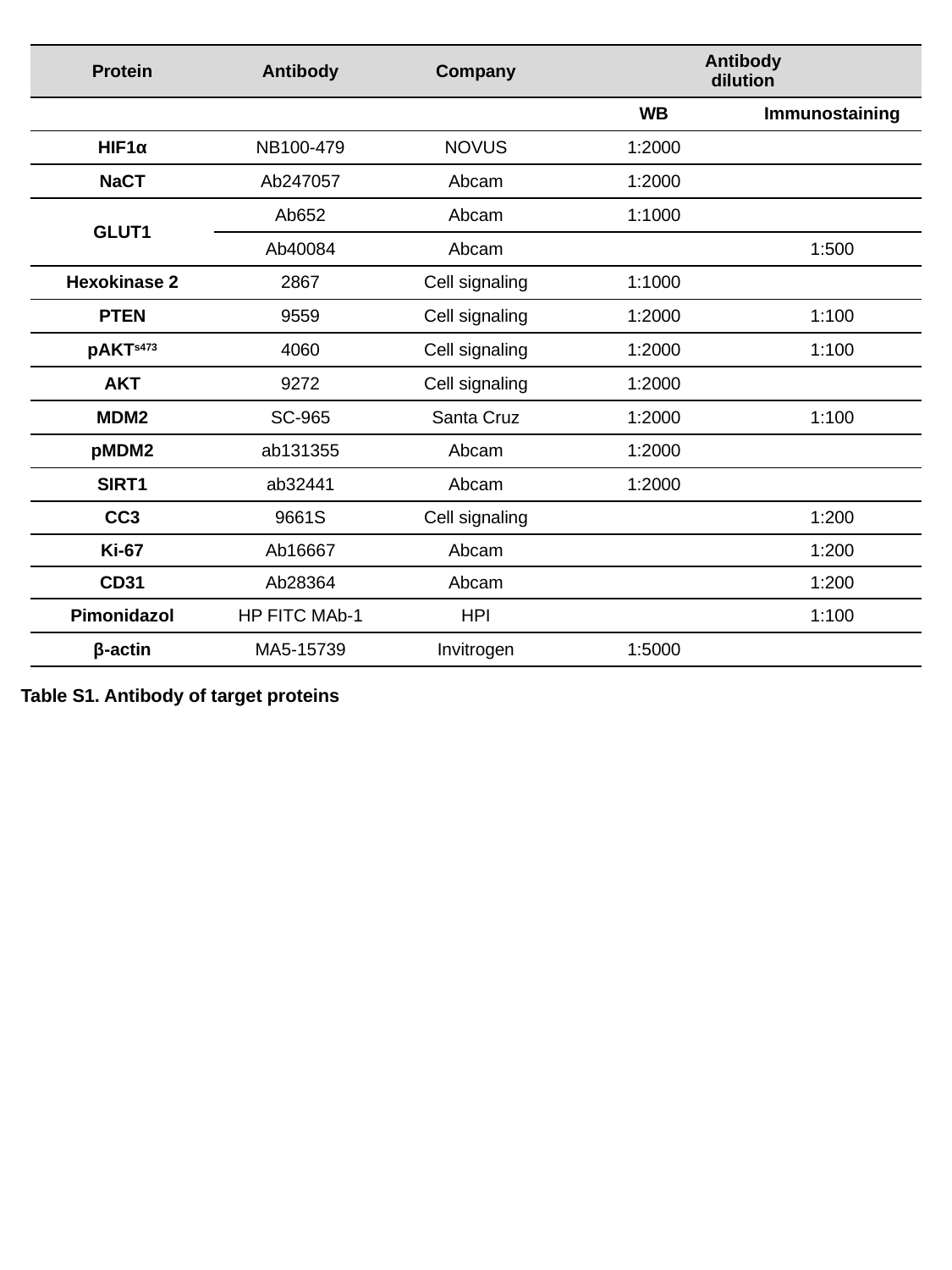

| Protein | Antibody | Company | Antibody dilution | |
| --- | --- | --- | --- | --- |
| | | | WB | Immunostaining |
| HIF1α | NB100-479 | NOVUS | 1:2000 | |
| NaCT | Ab247057 | Abcam | 1:2000 | |
| GLUT1 | Ab652 | Abcam | 1:1000 | |
| | Ab40084 | Abcam | | 1:500 |
| Hexokinase 2 | 2867 | Cell signaling | 1:1000 | |
| PTEN | 9559 | Cell signaling | 1:2000 | 1:100 |
| pAKTs473 | 4060 | Cell signaling | 1:2000 | 1:100 |
| AKT | 9272 | Cell signaling | 1:2000 | |
| MDM2 | SC-965 | Santa Cruz | 1:2000 | 1:100 |
| pMDM2 | ab131355 | Abcam | 1:2000 | |
| SIRT1 | ab32441 | Abcam | 1:2000 | |
| CC3 | 9661S | Cell signaling | | 1:200 |
| Ki-67 | Ab16667 | Abcam | | 1:200 |
| CD31 | Ab28364 | Abcam | | 1:200 |
| Pimonidazol | HP FITC MAb-1 | HPI | | 1:100 |
| β-actin | MA5-15739 | Invitrogen | 1:5000 | |
Table S1. Antibody of target proteins
